# Pertussis infection in fully vaccinated children in day-care centers, Israel.

**DOI:** 10.3201/eid0605.000512

**Published:** 2000

**Authors:** I Srugo, D Benilevi, R Madeb, S Shapiro, T Shohat, E Somekh, Y Rimmar, V Gershtein, R Gershtein, E Marva, N Lahat

**Affiliations:** Department of Clinical Microbiology, Bnai Zion Medical Center, Haifa, Israel. srugoi@tx.technion.ac.il

## Abstract

We tested 46 fully vaccinated children in two day-care centers in Israel who were exposed to a fatal case of pertussis infection. Only two of five children who tested positive for Bordetella pertussis met the World Health Organization's case definition for pertussis. Vaccinated children may be asymptomatic reservoirs for infection.


### Pertussis Infection in Fully Vaccinated Children in Day-Care Centers, Israel

**Isaac Srugo,* Daniel Benilevi,* Ralph Madeb,* Sara Shapiro,† Tamy Shohat,‡ Eli Somekh,§ Yossi Rimmar,* Vladimir Gershtein,† Rosa Gershtein,* Esther Marva,¶ and Nitza Lahat†**
*Department of Clinical Microbiology, Bnai Zion Medical Center, Haifa, Israel; †Serology Laboratory, Carmel Medical Center, Haifa, Israel; ‡Israel Center for Disease Control, Tel Aviv, Israel; §Wolfson Medical Center, Tel Aviv, Israel; ¶Public Health Laboratories, Jerusalem, Israel

We tested 46 fully vaccinated children in two day-care centers in Israel who were exposed to a fatal case of pertussis infection. Only two of five children who tested positive for *Bordetella pertussis* met the World Health Organization's case definition for pertussis. Vaccinated children may be asymptomatic reservoirs for infection.

Pertussis, an acute disease of the upper respiratory tract caused by the gram-negative bacillus *Bordetella pertussis*, lasts 6 to 8 weeks and has three clinical stages. The initial (catarrhal) stage resembles a common cold with a mild cough. The second (paroxysmal) stage is characterized by episodes of repetitive coughing during a single expiration, followed by a sudden inspiration that generates the typical "whoop." The final (convalescent) stage, which lasts 1 to 2 weeks, marks a decrease in the severity and frequency of the cough (1).

Since the introduction of routine childhood vaccine, pertussis has been considered preventable, and pertussis-associated illness and deaths are uncommon (2). However, vaccine-induced immunity wanes after 5 to 10 years, making the vaccinated host vulnerable to infection (3). This susceptibility has been described in outbreaks of pertussis infection in highly vaccinated populations (3-6).

A recent study by Yaari et al. showed that infection in a vaccinated person causes milder, nonspecific disease, without the three classical clinical stages (7). Whooping cough is seen in only 6% of such cases; instead, the illness is characterized by a nonspecific, prolonged cough, lasting several weeks to months. Because of these atypical symptoms, pertussis infection is underdiagnosed in adults and adolescents, who may be reservoirs for infection of unvaccinated infants (8-10). In a study in France, up to 80% of infections in unvaccinated children were acquired from siblings and parents, suggesting that adults and even young siblings play a fundamental role in the transmission of pertussis (11).

Address for correspondence: Isaac Srugo, Department of Clinical Microbiology, Bnai Zion Medical Center, POB 4940, Haifa, Israel 31048; Fax: 972-4-835-9614; e-mail: srugoi@tx.technion.ac.il.

We demonstrated *B. pertussis* infection in fully vaccinated children ages 2-3 years and 5-6 years who had contact with an infected child. We investigated whether younger or recently vaccinated children may be protected from classical clinical illness but remain susceptible to infection and become asymptomatic carriers.

### The Study

We examined the family of a 4-month-old infant who died of pertussis in Israel, as well as children at two day-care centers that two siblings had attended during the infant's illness. The two siblings, ages 2 and 5 years, attended different day-care centers, for ages 2-3 years and 5-6 years, respectively. Both siblings continued to attend the centers despite paroxysmal cough for 4 to 5 weeks. Thirty other children attended the day-care center for the 2- to 3-year-old group. Sixteen other children attended the center for the 5- to 6-year-old group.

In the infant's family, a third sibling, age 11 years, also had a paroxysmal cough of 4 to 5 weeks' duration. The 35-year-old mother had a 3-month history of persistent cough. An 18-year-old aunt, who took care of the infant and lived in the same house, reported a mild respiratory illness without paroxysmal cough. None of the family members had a whooping episode, cyanosis, or pneumonia (Figure).

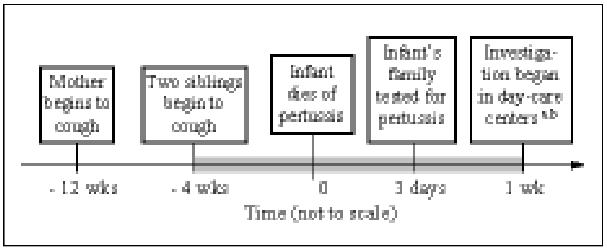

Figure. Timeline of pertussis infection in children in two day-care centers, Israel.

All the children in the day-care centers had been immunized in infancy with all four doses of Pasteur diphtheria-tetanus toxoid pertussis (DTP) vaccine, which includes a booster dose at 12 months of age. The Pasteur vaccine contains 1 immunization dose (ID) of purified diphtheria toxoid, 1 ID of purified tetanus toxoid, and >4 IU of *B. pertussis*. All family members of the infant were also fully vaccinated with four doses of DTP. The infant had received only the first dose of vaccine at 2 months of age.

The five family members of the infant and the 46 children in the two day-care centers were tested for *B. pertussis*. Two nasopharyngeal specimens were taken with Dacron swabs (Medical Wire, MEDECO, Corsham, UK); one specimen was used for culture and the other for polymerase chain reaction (PCR) testing. The culture specimen was immediately spread on charcoal agar plates (Hy Labs, Rehovot, Israel), which were incubated at 37°C for 14 days. Serum samples were also taken from every study participant for specific testing for immunoglobulin (Ig) M, IgA, and IgG antibodies to *B. pertussis* by an enzyme immunoassay (EIA) with whole-cell antigens (Panbio, East Brisbane, Australia) (12). Primers for the repeated insertion sequences were used in a semi-nested PCR assay (13-14). The upstream primer sequence gATTCAATA ggTTgTATgCATggTT and downstream primer AATTgCTggACCAT TCgAgTCgACG were used in the first PCR, which included 5 µL sample DNA, reaction buffer (10 mM Tris-HCl, 50 mM KCl, 1.5 mM $MgCl_2$, 0.1% Triton X-100), 1 µM of each primer, 200 µM deoxynucleotide triphosphate, and 1 U Taq polymerase (Boehringer Mannheim, Germany) in a 25-µL volume (14). Statistical analysis was performed by the two-tailed Fisher's exact test.

A person with positive PCR results was considered to have *B. pertussis* colonization of the nasopharynx. A person with positive IgM serum antibodies was considered to have had a recent infection. There were no culture-positive results, and nasopharyngeal aspirates were not available from the infant. Positivity by PCR or IgM did not indicate presence of symptoms.

Information on clinical symptoms was obtained from each person by a detailed questionnaire. The children in the day-care centers were followed clinically for 8 weeks after laboratory testing. All family members had been treated with erythromycin before testing, but no antibiotics were administered to the children in the day-care centers.

Eleven percent of the children in the two day-care centers were PCR positive, indicating nasopharyngeal colonization: 4 (25%) of the 16 5- to 6-year-old and 1 (3%) of the 30 2- to 3-year-old children ($p < .05$). Nine (55%) 5- to 6-year-old children were positive for serum IgM antibodies, and 4 (25%) were IgA positive. Three (10%) of the 2- to 3-year-old children were IgM positive, and 1 (3%) had IgA antibodies. Nasopharyngeal colonization was found more frequently in the 5- to 6-year-old than in the 2- to 3-year-old children (4/16 vs. 1/30, $p < .05$). This trend was also constant with IgM and IgA serum antibodies (9/16 vs. 3/30, $p < .001$ and 4/16 vs. 0/30, $p < .01$, respectively). In the index family, four of five members were positive by PCR, including all three siblings of the infant and the 18-year-old aunt. The 35-year-old mother, who was treated with erythromycin before testing, was negative by PCR. All five family members, including the mother, had high levels of IgM antibodies, indicating recent infection. The 4-month-old infant was seronegative for all subclasses of Ig antibodies to *B. pertussis*. No cultures were grown from the three groups.

According to a modified World Health Organization (WHO) case definition, two of the five children colonized with *B. pertussis* in the two day-care centers had the typical course of pertussis infection, with 3 weeks of paroxysmal cough (Table) (1). The other three children who were positive by PCR had only a mild, nonspecific cough during follow-up.

Table. Clinical and laboratory profiles of children positive for *Bordetella pertussis* by polymerase chain reaction (PCR) in Israel

| Day-Care Center | PCR+ | IgM[a]+ | IgA+ | Culture+ | Clinical Pertussis[b] |
|---|---|---|---|---|---|
| Ages 2-3 | | | | | |
| Child 1 | Yes | Yes | No | No | Yes |
| Ages 5-6 | | | | | |
| Child 2 | Yes | Yes | Yes | No | Yes |
| Child 3 | Yes | Yes | Yes | No | No[c] |
| Child 4 | Yes | Yes | Yes | No | No[c] |
| Child 5 | Yes | Yes | Yes | No | No[c] |

[a]Ig = Immunoglobulin.
[b]Paroxysmal cough >3 weeks; modified World Health Organization case definition (1).
[c]Nonspecific cough during 4 weeks of follow-up.

### Conclusions

The effects of whole-cell pertussis vaccine wane after 5 to 10 years, and infection in a vaccinated person causes nonspecific symptoms (3-7). Vaccinated adolescents and adults may serve as reservoirs for silent infection and become potential transmitters to unprotected infants (3-11). The whole-cell vaccine for pertussis is protective only against clinical disease, not against infection (15-17). Therefore, even young, recently vaccinated children may serve as reservoirs and potential transmitters of infection.

We used PCR, EIA, and culture to confirm *B. pertussis* infection in two highly vaccinated groups of children in two day-care centers. Three (10%) of 30 2- to 3-year-old children were seropositive for recent infection; one had nasopharyngeal colonization and a clinical illness that met the modified WHO case definition. In the day-care center for the 5- to 6-year-old group, 9 (55%) of 16 children were IgM positive, 4 (25%) of whom had nasopharyngeal colonization. Of these four children, three had nonspecific cough, and only one met the modified WHO definition for pertussis. None of the children in our study, including those who met the WHO definition, had been examined by a physician before our investigation.

Children who were seropositive and remained both asymptomatic and PCR negative probably had sufficient immunity from vaccines or natural boosters to protect them against persistent colonization and clinical disease. Their seropositivity could not be due to vaccine because the children were tested more than a year after having been vaccinated. Yet not all the children were protected from infection and from colonization with the bacteria. Whether a child who is serologically or PCR positive for pertussis and is clinically asymptomatic is a potential transmitter of infection has not been established. What is certain, however, is that vaccine-induced immunity against infection does not persist throughout adulthood. In France, booster vaccinations have been recommended for adolescents and teenagers (18). We found that immunity does not even persist into early childhood in some cases. We also observed that DPT vaccine does not fully protect children against the level of clinical disease defined by WHO. Our results indicate that children ages 5-6 years and possibly younger, ages 2-3 years, play a role as silent reservoirs in the transmission of pertussis in the community. More studies are needed to find the immunologic basis of protection against infection and colonization and thus an effective way to eradicate pertussis.

Dr. Srugo is a senior lecturer and director of the Clinical Microbiology and Pediatric Infectious Disease unit at the Bnai Zion Medical Center, Haifa, Israel.

### References

1. WHO meeting on case definition of pertussis: Geneva 10-11 January, 1991. Geneva: World Health Organization, 1991:4-5 (issue no. MIN/EPI/PERT/91.1)
2. Cherry JD. The epidemiology of pertussis and pertussis immunization in the United Kingdom and the United States: a comparative study. Curr Probl Pediatr 1984;14:1-78.
3. Jenkinson D. Duration of effectiveness of pertussis vaccine: evidence from 10-year community study. BMJ 1988;296:612-4.
4. Christie CD, Marx ML, Marchant CD, Reising SF. The 1993 epidemic of pertussis in Cincinnati: resurgence of disease in a highly immunized population of children. N Engl J Med 1994;331:16-21.
5. Rosenthal S, Strebel P, Cassiday P, Sanden G, Brusuelas K, Wharton M. Pertussis infection in young adults during the 1993 outbreak in Chicago. J Infect Dis 1995;171:1650-2.
6. De Melker HE, Conyn Van Spaendonck MA, Rumke HC, van Wijngaarden JK, Mooi FR, Schellekens JF. Pertussis in the Netherlands: an outbreak despite high levels of immunization with whole-cell vaccine. Emerg Infect Dis 1997;3:175-8.
7. Yaari E, Yafe-Zimerman Y, Scwartz SB, Slater PE, Shvartzman P, Andoren N, et al. Clinical manifestations of *Bordetella pertussis* infection in immunized children and young adults. Chest 1999;115:1254-8.
8. Aoyama T. Takeuchi Y, Goto A, Iwai H, Murase Y, Iwata T. Pertussis in adults. Am J Dis Child 1992; 146:163-6.

9. Cromer BA, Boydos J, Hackell J, Mezzatesta J, Dekker C, Mortimer EA. Unrecognized pertussis infection in adolescents. Am J Dis Child 1993;147:575-7.
10. Nelson JD. The changing of epidemiology of pertussis in young infants: the role of adults as reservoirs of infection. Am J Dis Child 1978;132:371-3.
11. Baron S, Njamkepo E, Grimprel E, Begue P, Desenclos JC, Drucker J, et al. Epidemiology of pertussis in French hospitals in 1993 and 1994: thirty years after a routine vaccination. Pediatr Infect Dis J 1998;17:412-8.
12. He Q, Mertsola J, Soini H, Skurnik M, Ruuskanen O, Viljanen MK. Comparison of polymerase chain reaction with culture and enzyme immunoassay for diagnosis of pertussis. J Clin Microbiol 1993;31:642-5.
13. He Q, Mertsola I, Soini H, Viljanen MK. Sensitive and specific polymerase chain reaction assays for detection of *Bordetella pertussis* in nasopharyngeal specimens. J Pediatr 1994;124:421-6.
14. Lichtinghagen R, Diedrich-Glaubitz R, von Horsten B. Identification of *Bordetella pertussis* in nasopharyngeal swabs using a polymerase chain reaction: evaluation of detection methods. European Journal of Clinical Chemistry and Biochemistry 1994; 32:161-7.
15. Fine PEM, Clarkson JA. The recurrence of whooping cough: possible implications for assessment of vaccine efficacy. Lancet 1982;l:666-9.
16. Long SS, Welkon CJ, Clark JL. Widespread silent transmission of pertussis in families: antibody correlates of infection and symptomatology. J Infect Dis 1990;161:480-6.
17. Minh NNTM, He Q, Edelman K, Olander RM, Viljanen MK, Arvilommi H, et al. Cell-mediated immune response to antigens of *Bordetella pertussis* and protection against pertussis in schoolchildren. Pediatr Infect Dis J 1999;18:366-70.
18. Grimprel E, Baron S, Levy-Bruhl D, Garnier JM, N'jamkepo E, Guiso N, et al. Influence of vaccination coverage on pertussis transmission in France. Lancet 1999;354:1699-700.
